# Surveillance and genetic diversity analysis of avian astrovirus in China

**DOI:** 10.1371/journal.pone.0264308

**Published:** 2022-02-28

**Authors:** Fuyou Zhang, Yang Li, Wenming Jiang, Xiaohui Yu, Qingye Zhuang, Suchun Wang, Liping Yuan, Kaicheng Wang, Shuhong Sun, Hualei Liu

**Affiliations:** 1 Shandong Agricultural University, Tai’an, Shandong, China; 2 China Animal Health and Epidemiology Center, Qingdao, Shandong, China; Foshan University, CHINA

## Abstract

Avian astroviruses (AAstVs) have caused major problem for poultry breeding industries in China in recent years, and the goose gout caused by goose astrovirus has produced particularly great economic losses. To better understand the prevalence and genetic diversity of AAstVs in China, 1210 poultry samples collected from eight provinces were tested with reverse transcription-polymerase chain reaction (RT-PCR) to detect AAstV infections in different poultry populations. Then, Open reading frames 2 (ORF2) was amplified by specific primers, and the genetic evolution was analyzed. Our surveillance data demonstrate the diversity of AAstVs in China insofar as we detected 17 AAstVs, including seven chicken astroviruses (CAstVs), five avian nephritis viruses (ANVs), two goose astroviruses (GoAstVs), two duck astrovirus (DAstVs), and one new AAstV belonging to *Avastrovirus Group 3*. The positive rate of AAstV infection was 1.40%. Host analysis showed that CAstVs and ANVs were isolated from chickens, DAstVs and GoAstVs were isolated from ducks. Host-species-specific AAstVs infections were also identified in numerous samples collected at each stage of production. This study provides further evidence to better understand the epidemiology of AAstVs in different species of poultry in China.

## Introduction

Avian astroviruses (AAstVs), which belongs to the genus *Avastrovirus*, are single-stranded positive-sense RNA viruses that lack a capsule. The virus was first identified in ducks in 1965 [1]. The AAstV genome length is 6.1–7.9kb, and it contains three open reading frames (ORFs: ORF1a, ORF1b and ORF2), a 5′ untranslated region (UTR), a 3′ UTR, and a poly (A) tail [2]. The family *Astroviridae* contains two genera, *Mamastrovirus* and *Avastrovirus* (AAstV), which consist of astroviruses that infect mammalian and avian species, respectively [3, 4]. The classification within each genus was previously based on the host of origin [5], but a limitation of this system is that the species thus defined do not correspond to genetic phylogenies. Therefore, *Astrovirus* species are currently defined on the basis of genetic differences in the complete capsid sequence.

According to the International Committee of Taxonomy of viruses (ICTV) in the latest 2019 edition, the genus AAstV containing three species: Avastrovirus 1 (AAstV-1) including turkey astrovirus 1 (TAstV-1), Avastrovirus 2 (AAstV-2) including avian nephritis virus 1 (ANV-1), ANV-2, ANV-3 and chicken astrovirus (CAstV), and Avastrovirus 3 (AAstV-3) including turkey astrovirus 2 (TAstV-2), turkey astrovirus 3 (TAstV-3) and duck astrovirus 1 (DAstV-1) [5–8]. As well as the three officially approved *Avastrovirus* species, there are many other avastroviruses yet to be classified, such as guineafowl astrovirus (GFAstV), goose astrovirus (GoAstV), duck hepatitis virus type 3 (DHV-3) [9], DHV-3-like astroviruses [10], duck astrovirus CPH (DAstV CPH) [11], and astroviruses detected in wild birds [12].

In recent years, AAstV infections have been shown to be associated with many diseases [9] that pose a threat to the poultry breading industries. These include poultry enteritis mortality syndrome, runting-stunting syndrome in broilers, kidney and visceral gout in broilers, and fatal hepatitis in ducklings [13, 14]. A previous study reported that the antibody positive rate for CAstV in chickens in China is 60.68% [15]. Astroviruses infections are becoming more common in China, including those of ANV, CAstV, DAstV, and GoAstV [12, 16–18]. Some astrovirus isolates have caused fatal visceral gout in domestic goslings [19]. These studies suggest that more attention must be paid to astrovirus infections in poultry.

In the present study, we undertook an epidemiological investigation in 2020 with the routine surveillance of AAstVs using universal RT-PCR, to better understand the prevalence and distribution of AAstVs in China. Our results demonstrate the genetic diversity of AAstVs in China, and extend the epidemiological information available on these viruses.

## Materials and methods

### Ethics statement

This study was conducted according to the Animal Welfare Guidelines of the World Organization for Animal Health [20] and was approved by the Animal Welfare Committee of the China Animal Health and Epidemiology Center (CAHEC, Qingdao, Shandong).

### Sample collection

A total of 1210 swab and tissue samples were collected from 20 regions in eight provinces of China in 2020 (the sample background information was shown in **Table 1**), mainly for routine surveillance of avian diseases, such as avian influenza virus (AIVs) and Newcastle disease virus (NDVs) infection, among others. The swab and tissue samples were collected by taking smears from both the cloacal and oropharyngeal tracts of the poultry, and were stored in 1.5 mL of phosphate-buffered saline (pH 7.2) containing 10% glycerol. The RNA was extracted with the RNeasy Mini Kit (Qiagen, Hilden, Germany), according to manufacturer’s instructions, and stored at -80°C.

**Table 1:** Background information of the samples.

Province	Time	Host	Sample type
Guangdong	2020	chicken、duck、goose	Swab
Sichuan	2020	chicken、duck、goose	Swab
Fujian	2020	duck、chicken	Swab
Guangxi	2020	duck、chicken	Swab
Henan	2020	duck、chicken	Swab
Jiangsu	2020	chicken、duck、goose、pigeon	Swab
Heilongjiang	2020	chicken、duck、goose	Swab
Shandong	2020	duck、chicken	Swab、Tissue

### Detection of AAstVs with conserved RT-PCR assay

Conserved RT-PCR and sequencing were used to analyze the genetic characteristics of the AAstVs circulating in poultry. In brief, the RNAs described above were amplified with the Takara One Step RT-PCR Kit (Takara), using primers to conserved sites in the AAstV genomes: 5’-GAYTGGACNMGNTWYGAYGGNACNATNCC-3’ (forward) and 5’-YTTNACCCACATNCCRAA-3’ (reverse) [21]. The assay amplified a 430-bp region of the viral RdRp, which has been shown to detect all astroviruses circulating in avian species, including chickens, ducks, and geese. The RT-PCR assay was performed in a 25μL reaction system, which was incubated at 50°C for 30 min and then denatured at 94°C for 5 min, followed by 45 cycles of 94°C for 1 min, 45°C for 1 min and 72°C for 40 s. The PCR products were purified by DNA extraction kit (Sangon, Shanghai, China), and sequenced directly with the ABI 3730xl DNA Analyzer. Then, the primers in the **Table 2** are used to amplify ORF2 (Amplification primers are shown in **Table 2**). RT-PCR detection was performed in a 25μL reaction system, which was incubation at 50°C for 30 min and then denatured at 94°C for 5 min, followed by 45 cycles of 94°C for 1 min, 45°C for 1 min and 72°C for 1 min 30 s. The PCR products were purified and sequenced, and the sequence obtained by sequencing is used to construct a phylogenetic tree.

**Table 2:** Amplification primers of AAstV isolate ORF2.

Name	Primer sequence	Tm value	Fragment size	Position
L1389-F1	GAAGACATACAGTTACCAGAAG	50	1197	4915–6111
L1389-R1	CGAGGCATACACAGCATAA			
L1389-F2	TCCACTACGGTTGTTGATG	50	1052	5980–7031
L1389-R2	CTACACTGCTCCTTCCAATA			
J1018-F1	AATACGCTCGTGCTGAAG	50	1216	4481–5696
J1018-R1	GGAGAGTAATCTGTGTTGTTC			
J1018-F2	ACTGCTCTCGTTACTCAC	50	1232	5422–6653
J1018-R2	AAGTTAGTTGCTGTAGTCAG			
J1013-F1	CACTCAACACTGGTTCTCA	50	1213	5596–6808
J1013-R1	GGAGCGTAGTTCTCAACAG			
J1013-F2	CTGCCAGACCTTGAATCC	50	1242	4373–5614
J1013-R2	TGAGAACCAGTGTTGAGTG			
616G1-F1	CAAGGTCTCTGATGATATTGAG	50	1466	4500–5965
616G1-R1	TCGGTGGAACTGTCTGAT			
616G1-F2	AGGAGGATGGTTCGTGAT	50	1208	5841–7048
616G1-R2	CGGGTTAATTTAAGGCAGAG			
Y-5-F1	ATGCTCCAGAGAATGTTAGG	50	1348	5165–6512
Y-5-R1	ATTGGTTAGTTGCGGTGAA			
Y-5-F2	GCTACTTGGTGTATTCTTCAG	50	1230	6427–7656
Y-5-R2	GCGATATGCCAGGTTGAT			
G1254-F1	CCGACAATGTTAGAGACTTC	50	1109	4786–5894
G1254-R1	TGAGACTGACCAGAATGTG			
G1254-F2	AAGTGTGCTTGAAGTTGATG	50	1260	5819–7078
G1254-R2	CTGCTGGTTGAAGGTGTAT			
G1272-F1	GGGAGCGTAGGTTTGATAG	50	1251	128–1378
G1272-R1	CACAATAGGCACTGAGGTT			
G1272-F2	AAGAGAATGCTACTGATGAGG	50	888	1004–1891
G1272-R2	GGGTTCCAAATGGTTGAATAG			
J1002-F1	GTTCTCGCTCTCGTTCAA	50	1498	5072–6569
J1002-R1	GCTGTATGTATCACTCCAATG			
J1002-F2	CATTGAGGACGCCAACAA	50	1307	6126–7432
J1002-R2	CGATAGACCTGATTGACACA			
H1283-F1	AGTTGGATAATCACGAGAGG	50	1127	5173–6299
H1283-R1	TGGCAGGTAGTCAGTTGT			
H1283-F2	TGAGGACGCCAATAAGGA	50	1022	6140–7161
H1283-R2	GGACAAGAGGAGTATTAGCA			

### Phylogenetic analysis

The ORF2 sequences were aligned, and the substitutions and phylogenetic relationships were calculated with the software package MEGA 6.0 [22, 23]. The phylogenetic relationships of the sequences were calculated with the maximum likelihood method, which is assumed to best describe the substitution pattern. Gaps were handled by partial deletion, and bootstrap values were calculated from 1000 replicates.

## Results

### Distribution of the samples

**Fig 1:**
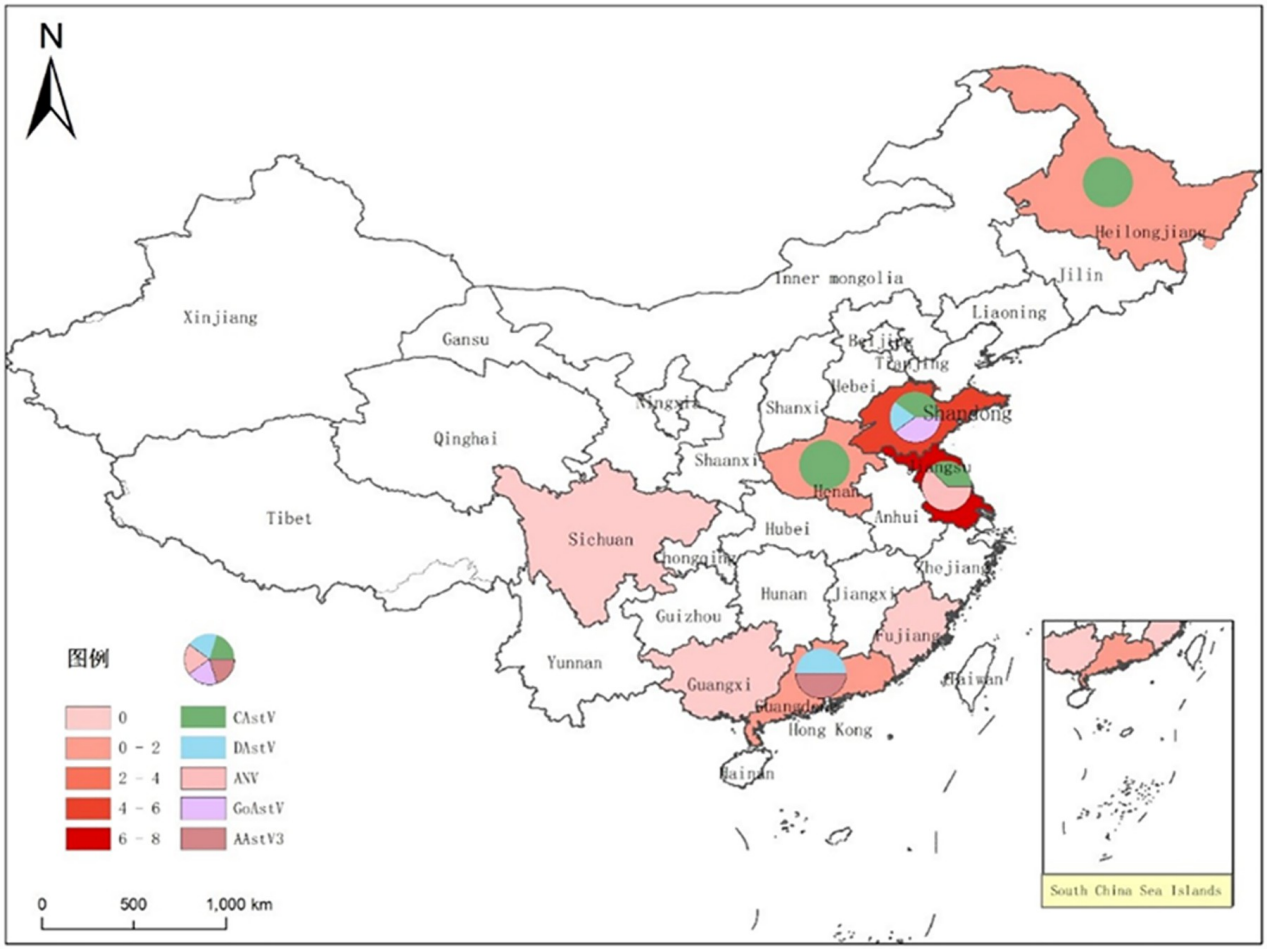
Geographical distribution of the samples collected. Map was created using ArcGIS by ESRI version 10.8 (http://www.esri.com). Map source data was obtained from the Natural Earth (http://www.naturalearthdata.com/).

A total of 1210 swabs and tissue samples were collected in eight provinces of China (Guangdong, Guangxi, Sichuan, Fujian, Henan, Jiangsu, Heilongjiang and Shandong), and included 1160 swabs and 50 tissues. The geographic distribution of the samples analyzed in this study is shown in **Fig 1**. All the samples, including 435 chicken samples, 666 duck samples, 5 pigeon samples, 104 geese samples, originated from 22 retail markets, 6 wholesale markets, 2 slaughterhouse, and 20 poultry farms.

### Surveillance of *Astrovirus* circulating in poultry

Seventeen samples tested positive for AAstV with RT-PCR and the PCR products were sequenced. The accession numbers deposited in GenBank for the sequences of the 17 Astroviruses are MZ097383-MZ097399 (**Table 3**).

**Table 3:** The information of astrovirus identified in this study.

Strain	Years	Province	Host	Species	GenBank Accession
0607	2020	Shandong	Chicken	Chicken Astrovirus	MZ097383
N-2P	2019	Shandong	Chicken	Chicken Astrovirus	MZ097384
J1002	2020	Jiangsu	Chicken	Chicken Astrovirus	MZ097385
J1011	2020	Jiangsu	Chicken	Chicken Astrovirus	MZ097386
J1049	2020	Jiangsu	Chicken	Chicken Astrovirus	MZ097387
H1283	2020	Henan	Chicken	Chicken Astrovirus	MZ097388
L1389	2020	Heilongjiang	Chicken	Chicken Astrovirus	MZ097389
J1013	2020	Jiangsu	Chicken	Avian nephritis virus	MZ097390
J1017	2020	Jiangsu	Chicken	Avian nephritis virus	MZ097391
J1018	2020	Jiangsu	Chicken	Avian nephritis virus	MZ097392
J1038	2020	Jiangsu	Chicken	Avian nephritis virus	MZ097393
J1054	2020	Jiangsu	Chicken	Avian nephritis virus	MZ097394
Y-5	2020	Shandong	Duck	Duck Astrovirus	MZ097395
G1254	2020	Guangdong	Duck	Duck Astrovirus	MZ097396
G1272	2020	Guangdong	Duck	Avastrovirus 3	MZ097397
616g1	2020	Shandong	Duck	Goose Astrovirus	MZ097398
616g2	2020	Shandong	Duck	Goose Astrovirus	MZ097399

### Phylogenetic and diversity analyses of *Astrovirus* circulating in poultry

**Fig 2:**
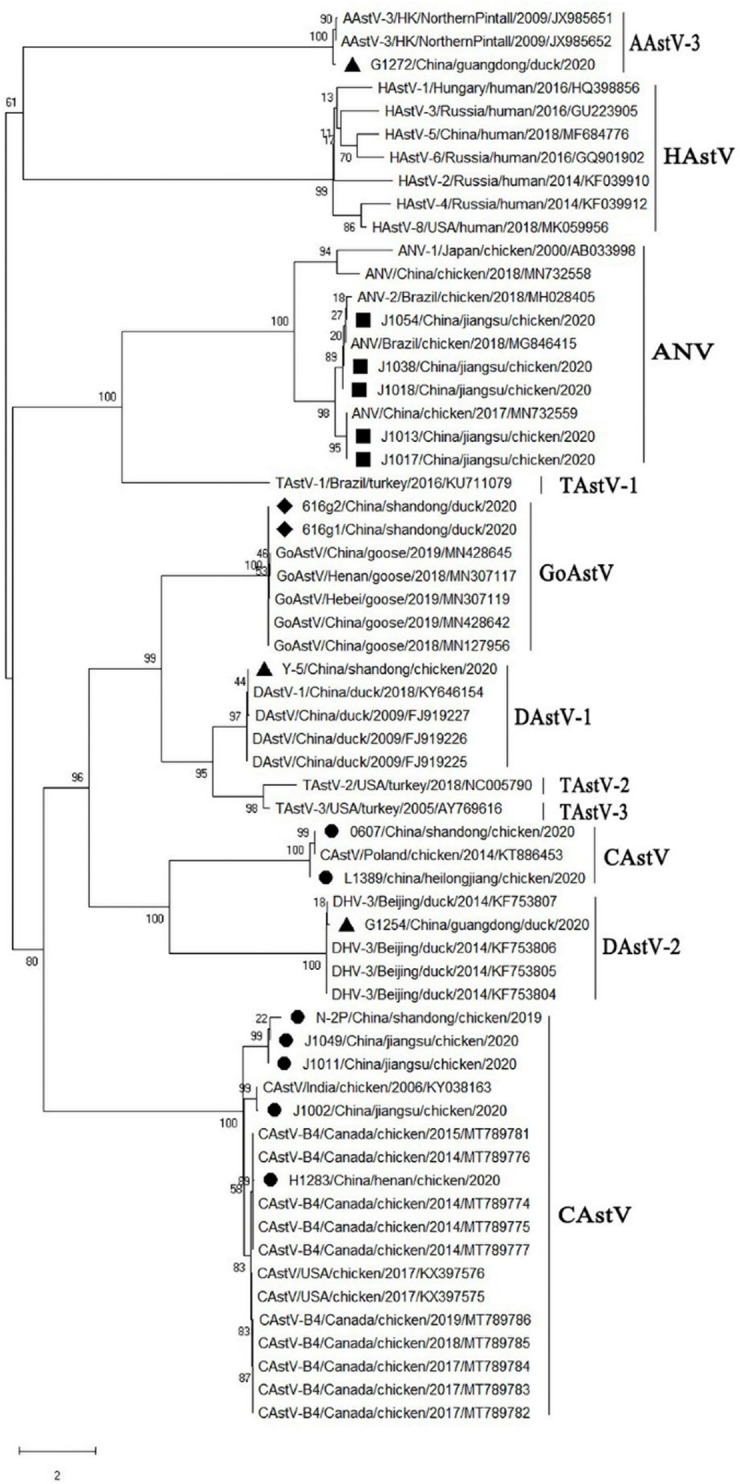
Phylogenetic trees based on the RdRp gene sequences of astrovirus. The trees were constructed using the model with the maximum likelihood (ML) method, gaps were handled by partial deletion and bootstrap values were calculated out of 1000 replicates. CAstV: chicken astrovirus; DAstV: duck astrovirus; ANV: avian nephritis virus; GoAstV: goose astrovirus. Seven CAstV representative strains were marked with yellow solid circles (●), one DAstV representative strains were marked with black hollow circle (▲), five ANV representative strains were marked with pink solid triangle (■), two GoAstV representative strains were marked with green solid diamond (♦), one new AAstV representative strains were marked with black hollow diamond (▲).

A phylogenetic analysis revealed that the 17 astroviruses detected in poultry could be classified into five lineages, and corresponded to seven CAstVs, five ANVs, two GoAstVs, two DAstVs, and one new AAstV belonging to *Avastrovirus Group 3* (**Fig 2**). 17 isolates were analyzed for homology with 43 strains downloaded from Genbank, and the results are shown in **Table 4**, and it is worth noting that the new AAstV isolate have high homology with the Hong Kong strain, which ranging from 92.6% to 92.8%.

**Table 4:** The results of homology analysis of ORF2 between 17 isolates and the reference strains.

Strain	Type	Host	ORF2
0607	CAstV	chicken	22.5%-98.8%
N-2P	CAstV	chicken	23.7%-84.6%
H1283	CAstV	chicken	17.7%-95.9%
L1389	CAstV	chicken	28.4%-94.7%
J1002	CAstV	chicken	28.7%-95.5%
J1011	CAstV	chicken	27.8%-97.6%
J1049	CAstV	chicken	27.9%-97.6%
616g1	GoAstV	duck	28.7%-99.5%
616g2	GoAstV	duck	28.7%-99.5%
Y-5	DAstV-1	duck	27.5%-99.9%
G1254	DHV-3	duck	29.0%-96.7%
G1272	AAstV-3	duck	28.7%-92.8%
J1013	ANV	chicken	27.4%-99.7%
J1017	ANV	chicken	27.4%-99.1%
J1018	ANV	chicken	63.8%-99.4%
J1038	ANV	chicken	27.1%-99.5%
J1054	ANV	chicken	27.0%-99.8%

The number of samples and the AAstV-positive rate in each province are shown in **Table 5**, and the geographical distribution of the samples detected in this study is shown in **Fig 1**. The rate of AAstV-positive samples was 1.40%, and the positivity rates of CAstV, ANV, DAstV, GoAstV and AAstV 3 were 0.66%, 0.41%, 0.08%, 0.17% and 0.08%, respectively. These data suggested that CAstV and ANV circulate in chickens, DAstV and GoAstV circulate in ducks. Two GoAstV strains were found in ducks when the host were analyzed (**Table 6**), whereas GoAstV was not detected in goose populations.

**Table 5:** Positive rate (%) of astrovirus in the samples collected from 8 provinces.

Province	Number of samples	CAstV	ANV	DAstV	GoAstV	AAstV 3	Total	Rate
Guangdong	142			1		1	2	1.41%
Sichuan	281						0	0
Fujian	282						0	0
Guangxi	102						0	0
Henan	58	1					1	1.72%
Jiangsu	192	3	5				8	4.17%
Heilongjiang	103	1					1	1.04%
Shandong	50	2		1	2		5	10.00%
Total	1210	7	5	2	2	1	17	1.40%

**Table 6:** Positive rates (%) of AAstV in the samples collected from four host species in 2020.

	CAstV	DAstV	ANV	GoAstV	AAstV 3	Rate
Chicken	7		5			2.76%(12/435)
Duck		2		2	1	0.75%(5/666)
Goose						0(0/104)
Pigeon						0(0/5)
Total	7	2	5	2	1	1.40%(17/1210)

Of the 1210 samples tested, 526 were collected from retail markets, 553 were from wholesale markets, 15 were from slaughterhouse, and 116 were from poultry farms, as shown in **Table 7**. Host-species-specific AAstV infections were identified in numerous samples collected at all the four types of poultry sites. Poultry farms had the highest positive rate, followed by the retail markets.

**Table 7:** Numbers of due-species infections and cross-species infections in the samples collected in 2020.

	CAstV	DAstV	ANV	GoAstV	AAstV 3	Rate
Retail Markets (n = 526)	4		5			1.71%
Wholesale Markets (n = 553)		1			1	0.36%
Slaughterhouse (n = 15)	1					6.67%
Poultry Farms (n = 116)	2	1		2		4.31%

## Discussion

DAstV was the first-discovered AAstV, in 1965, when it caused a case of viral hepatitis [1]. The virus was confirmed as DAstV-2 until 1985 [24]. In 2011–2012, an outbreak of gout was reported in broilers in India. After the isolation and identification of the virus, Bulbule confirmed that a new type of CAstV was one of the pathogens responsible for gout disease in chicken flocks in India [13]. In China, gout disease was first reported in 1-week-old goslings in Anhui province in 2015, after which the disease spread to most provinces in China in 2017, with a high incidence rate of 80%-90% and a mortality rate of 20%-70% [19].

Duck viral hepatitis is recognized as an acute, highly contagious, and fatal disease, for which there is no specific treatment. ANV and CAstV are associated with growth problems, kidney lesions and enteritis in young chickens, and CAstV infection has been shown to be prevalent in broilers. In addition to these three groups of DAstVs found primarily in domestic ducks, other AAstVs have also been identified in domestic ducks, including ANV, TAstV-1, TAstV-2 and CAstV [10, 25, 26]. The occurrence of different AAstVs in domestic ducks raises concern about the role of domestic ducks as reservoirs for diverse astroviruses. Previous studies have detected ANV in dead-in-shell goslings, suggests that attention must be paid to astrovirus infections in domestic geese [26].

AAstV are widely spread throughout the world and have become increasingly harmful to the poultry industry. However, their epidemiology and pathogenic mechanism are not yet clear. In this study, we conducted a surveillance of AAstVs with a conserved RT-PCR assay, which detected several different AAstVs, including CAstV, ANV, DAstV and GoAstV. Our results suggested that a diversity of astroviruses is present in poultry, including CAstVs, ANVs, DAstVs, GoAstVs, and novel AAstV.

Our results also suggested that AAstVs are present in many provinces of China, and that CAstV and ANV are circulating in chickens and DAstV and GoAstV in ducks. Interestingly, the two GoAstV strains were isolated from ducks in an analysis of their hosts, which suggests that CAstV, ANV and DAstV were all prevalent in their dominant hosts in 2020, whereas GoAstV was not detected in geese. This finding warrants further research.

In this study, 17 of AAstV were detected in 5 provinces of China, and nearly half of the strains were isolated from Jiangsu Province, This is consistent with the results of serological testing by Xue et al, which suggested that the epidemic of AAstVs in this areas was very serious [15]. Two CAstVs and two GoAstVs were detected in samples collected from a symptomatic poultry in Shandong province. This is consistent with the previous detection and identification of AAstVs in Shandong Province [16]. A novel AAstV was detected in Guangdong Province, which was classified as *Avastroviruses Group 3*, but its pathogenicity in poultry is still unclear and requires further research. The results of this study suggest that other new astroviruses are yet to be found.

Of the 1210 samples tested, host-species-specific AAstV infections were identified in samples collected from many different poultry populations. Therefore, each stage of the production process, from breeding to marketing to slaughter (in poultry farms, live poultry markets, and slaughterhouses) may play important roles in the circulation of AAstVs in poultry, as they do in the circulation of AIVs.

To our knowledge, this study is the first to report a systematic phylogenetic analysis of AAstV strains in China. The data presented extend the epidemiological information available on AAstVs in China. However, the continuous surveillance of avastrovirus should be increased to better understand the diversity, distribution, cross-species transmission and clinical significance of AAstVs in nature.

## Supporting information

S1 Table Relevant data for geographic distribution maps. A total of samples collected from 8 provinces were tested (column ID = “NameEN”). The number of positives detected in each province ranges from 0 to 8 (column ID = “Total”), The positive rate varies from 0–10% (column ID = “Rate”).
(DOCX)
