# Supplementary material for: Surveillance and genetic diversity analysis of avian astrovirus in China
Source: PLoS One. 2022 Feb 28;17(2):e0264308. doi: 10.1371/journal.pone.0264308 (PMC8884486; doi:10.1371/journal.pone.0264308)
Supplement: S1 Table — A total of samples collected from 8 provinces were tested (column ID = “NameEN”). The number of positives detected in each province ranges from 0 to 8 (column ID = “Total”), The positive rate varies from 0–10% (column ID = “Rate”). (DOCX) [file pone.0264308.s001.docx]

**S1 Table. Relevant data for geographic distribution maps.**

| **NameEN** | **CAstV** | **DAstV** | **ANV** | **GoAstV** | **AAstV 3** | **Total** | **Rate** |
| --- | --- | --- | --- | --- | --- | --- | --- |
| **Guangdong** | 0 | 1 | 0 | 0 | 1 | 2 | 1.41% |
| **Sichuan** | 0 | 0 | 0 | 0 | 0 | 0 | 0 |
| **Fujian** | 0 | 0 | 0 | 0 | 0 | 0 | 0 |
| **Guangxi** | 0 | 0 | 0 | 0 | 0 | 0 | 0 |
| **Henan** | 1 | 0 | 0 | 0 | 0 | 1 | 1.72% |
| **Jiangsu** | 3 | 0 | 5 | 0 | 0 | 8 | 4.17% |
| **Heilongjiang** | 1 | 0 | 0 | 0 | 0 | 1 | 0.97% |
| **Shandong** | 2 | 1 | 0 | 2 | 0 | 5 | 10.00% |
| **Total** | 7 | 2 | 5 | 2 | 1 | 17 | 1.40% |
